# Chemical Examination of Mr. Smith's Flexible Metallic Bougies

**Published:** 1802-03

**Authors:** F. N. Vandier

**Affiliations:** No. 60, Great Mary-le-Bone Street


### *Chemical Examination of Mr.* SMITH's *Flexible Metallic Bougies.*

To the Editors of the MEDICAL and PHYSICAL JOURNAL.

Gentlemen,

I Had totally forgot that you announced the flexible Metallic Bougies in the fecond volume of the Medical and Phyfical Journal, but I have read lately fome account of their effects, and I was led to believe that fome confiderable improvement had been made in the treatment of a direful difeafe.

I am well aware that this is merely a revival of an ancient practice, and that Metallic Bougies have long been ufed in Surgery; abundant proofs of that fact may be found in the works of the authors who have treated on the fubject. I know alfo that the bad confequences arifing from the frequently repeated introduction of fo hard a body in the urethra, the membrane of which is fo delicate, and perhaps the difficulty of adapting to the urinary paffage a fubftance which, however foft and pliable, bends always at a more or lefs confiderable angle, had induced furgeons to have recourfe to the much milder means recommended by Daran, Goulard, and others.

Conceiving, notwithftanding, that every method of treating fo melancholy a difeafe as ftrictures in the urethra ought to be candidly examined, I procured fome of thefe Bougies, and I determined to fend fome to my valuable friend Francis Charoy, Surgeon in Chief to the eighth regiment of Huffards, garrifoned at Haguenau, in the department of the Lower Rhine. But before fending them, I thought it might not be altogether ufelefs to enquire into their compofition. A powerful incentive with me was, my being a decided enemy to any fecret in medicine, perfuaded as I am, that fecrefy is too often the cloak of empiricifm. If a remedy is fanctioned by repeated trials, a humane and generous practitioner will make it as public as poffible, that every fuffering individual may be benefited by it; if it has been propofed upon a fanciful hypothefis, as is often the cafe, even in this enlightened age, let it be rejected or admitted, mited, as experience will direct after a fair trial. Had the immortal Jenner kept his admirable difcovery a fecret, mankind would have continued to fall victims to that dreadful fcourge, the Small-pox.

In order, then, to contribute my fhare in the publicity of what I conceive to be an improved mode of treating ftrictures, I inftituted fome experiments which I fhall here relate, as well as the inductions to which they conducted me. I will not ftop here to defcribe the external appearance of thefe bougies, as they have nothing peculiarly remarkable, and as I fuppofe that moft medical gentlemen are acquainted with them. They are fmooth, firm, and poffefs a great degree of pliability. Thofe which I examined were of the largeft dimenfions (No. 11 and 12), they were nearly as flexible as common bougies of the fame fize. They were fold to me under the title of *Solid flexible Metallic Bougies.* Upon cutting them acrofs, they were found *hollow*, their internal furface was very bright, and the ends were fkilfully clofed. I muft here confefs, that I am not fufficiently verfed in the Englifh language to determine whether hollow tubes, ftopped at both ends, may with propriety be called folid bodies.

*Experiments with Nitric Acid.*

1. One hundred grains were put in a Florence flafk with concentrate nitric acid, upon a fand bath; an abundant difengagement of nitrous gas took place, and a white matter remained at the bottom. This powder was carefully feparated by filtration from the fupernatant liquor.

2. Sulphuric acid and fulphates were dropped in fome of the liquor. No precipitate of any kind was perceived. Hence no lead was prefent.

3. Water impregnated with fulphurated hydrogen gas caufed no precipitate. This excluded at once filver, mercury, copper, lead, tin, antimony, bifmuth, and arfenic.*

4. Carbonate of foda, dropped in the liquor, caufed no precipitate; therefore no metal was prefent.

5. Gold and platina I did not expect to find in the white precipitate, and the following experiments fhewed they ought not to be looked for.

*Experiments*

* I prefer the words, *water impregnated with fulphurated hydrogen gas* to *hydrofulphurated water*, or *water impregnated with fulphurated hydrogen gas*, for reafons which will foon be publifhed in "A Differtation upon Chemical Nomenclature, by Rd. Chenevix, Esq. F. R. S. M. R. I. A." The fame obfervation applies to *oxidifed* inftead of *oxidated*.

*Experiments with Muriatic Acid.*

1ſt. One hundred grains, placed as in the former experiments, but with diluted muriatic inſtead of nitric acid, were entirely diſſolved in a moderate ſpace of time with a diſengagement of hydrogen gas.

2d. The liquor could contain no other metals but thoſe which are capable of decompounding water, v. g. iron, tin, zinc, manganeſe. But there was no iron, for ammonia, Pruſſiate of ammonia, ſpirituous tincture of galls, cauſed no precipitate.

3d. That there could exiſt neither zinc nor manganeſe, the foregoing experiments with nitric acid (Exp. 4) had ſufficiently demonſtrated.

4th. When a drop of liquid muriate of gold was put in the liquor, a beautiful purple precipitate was formed, which is ſo well known under the name of Caſſius's precipitate. Hence tin was preſent.

It would be too long to detail all the experiments I have made. Some pure tin was oxydiſed by means of nitric acid, in order to ſerve as a point of compariſon; ſome was diſſolved in muriatic acid, and precipitated by carbonate of ſoda; and the reſult of all theſe experiments correſponded, as nearly as ſuch chemical trials will allow, with thoſe made with the bougies.

I was led to queſtion, in ſome meaſure, thoſe experiments, as I could not hear, on bending the bougies, that peculiar noiſe ſo well known, and called by the French *le cri de l'étain*; but upon preſſing them between the teeth, a ſenſible noiſe was eaſily perceived.

One hundred grains of the bougies were melted in a Heſſian crucible, and caſt into a mould; the ſurface was not leſs brilliant than the original bougie, but the ingot was leſs flexible on account of its *solidity*. It ſeemed to reſemble exactly common tin, and gave the uſual ſound when bent.

From the foregoing experiments it would appear to me, that the flexible metallic bougies are tin, and that they owe their pliability, as well as their want of ſound, to their being hollow. It will appear evident, I believe, that, if chemical reagents can be depended on, they are not a compound metal.

I was much aſtoniſhed, therefore, at the author's claim of *invention*. I am not well acquainted with the ancient hiſtory of Great Britain, but I well know that ſome authors derive the name of Britain from *Bratanac*, which is ſaid to ſignify, in the Phenician language, the *Country of Tin*; becauſe theſe people uſed to come particularly into Cornwall to buy that metal. They ſold it in their turn to the Greeks, who, on that account, called theſe fortunate iſlands *Caſſiterides*, from Κασσίτερος, Tin.

Indeed,

Indeed, this claim of *invention* appears to me to place the claimant in the fame predicament as a failor, who on going to fea for the firft time, fhould difcover the Ifle of Wight, or of a citizen of London, who beginning his travels, fhould difcover Brentford.

I fhould be very happy, Gentlemen, to prefent you with a chemical fynthefis of thefe bougies; but Nature has fecrets which fhe imparts to very few, and, for the prefent, I am afraid we fhall be obliged to reft fatisfied with the poffibility of obtaining from the mines of Cornwall, the metal of which they are formed. As to the mechanical conftruction, we can obtain information from every pewterer.

With refpect to their utility as bougies or catheters in furgery, I will leave it to furgeons to determine; my fole object was to examine whether they were a compound metal, or had any claim to a difcovery.

*No.* 60, *Great Mary-le-Bone Street,* *February* 11, 1802.

I am, &c.
F. N. VANDIER.

P. S. I take the liberty of enclofing the following paffage, as it may tend to elucidate important facts, and as probably few of your readers fee the Journal from which it is tranflated.

"Citizen Texier, Surgeon in Chief to the Military Hofpital of Invalids at Verfailles, has continued the experiments which he had undertaken in concert with Citizens Alibert, Balfac, and Valois, with a view to determine whether the infertion of the Vaccine virus would guard fheep againft the fcab.* The refults which he obtained lately, deferve being publifhed; he inoculated with the virus of the fcab two fheep which had been previoufly vaccinated, and neither was found fufceptible of the contagion. Every one will perceive how interefting it is, for the improvement of rural economy, to collect fimilar facts, and efpecially how important it is to repeat and multiply the trials." Décade Philofophique, An. x. No. 10.

---

* Malis Cornipedum of Liger, la Clavelée or le Claveau; a difeafe very frequent among fheep, and which is faid to be contagious; it confifts in boils or furuncles which infeft nearly the whole body. Worms are found in the cavity of thefe boils, when they come to fuppuration; thefe worms come from the eggs depofited there by infects, which often find means to creep and penetrate alfo into the nofe and frontal finufes. See Sauvages, Clafs x. Gen. Malis.

